# Pharmacological investigation of brucine anti-ulcer potential

**DOI:** 10.3389/fphar.2022.886433

**Published:** 2022-08-17

**Authors:** Muhammad Noman, Neelum Gul Qazi, Najeeb Ur Rehman, Arif-ullah Khan

**Affiliations:** ^1^ Riphah Institute of Pharmaceutical Sciences, Riphah International University, Islamabad, Pakistan; ^2^ Department of Pharmacology and Toxicology, College of Pharmacy, Prince Sattam Bin Abdulaziz University, Al-Kharj, Saudi Arabia

**Keywords:** brucine, anti-ulcer, anti-*H. pylori*, H^+^/K^+^-ATPase, antioxidant, anti-inflammatory

## Abstract

Gastric ulcer is one of the most common chronic gastrointestinal diseases characterized by a significant defect in the mucosal barrier. The current study has been conducted to evaluate the brucine anti-ulcer effect. Brucine has binding energy values ranging from −2.99 to −8.11 kcal/mol against chosen targets, according to *in silico* research. Brucine exhibits an inhibitory effect against *Helicobacter pylori*. *In vivo* findings revealed that brucine (3 mg/kg) showed effective results in healing ethanol-induced ulcer lesions of the gastric region in rats. Brucine showed an inhibitory effect against H^+^/K^+^-ATPase. Levels of glutathione, glutathione-s-transferase, and catalase were enhanced in the gastric rat tissue with the use of brucine, while a significant decrease in lipid peroxide levels was seen. Histopathological evaluation showed improvement in cellular architecture and a decrease in inflammatory indicators like cyclooxygenase, tumor necrosis factor, and nuclear factor kappa B expression, validated through immunohistochemistry, enzyme-linked immunosorbent assay, and Western blot techniques. In the reverse transcription–polymerase chain reaction, brucine decreased H^+^/K^+^-ATPase mRNA levels. This study reveals that brucine possesses stable binding affinities against selected targets. Brucine exhibits an anti-ulcer effect, mediated *via* anti-*H. pylori*, H^+^/K^+^-ATPase inhibition, and antioxidant and anti-inflammatory pathways.

## Introduction

Gastric ulcer is a disease characterized by secondary damage produced by excessive pepsin and stomach acid output. Frequent alcohol intake has a positive link to gastric mucosal lesions that include gastritis, gastric ulcer, and in severe conditions, also gastric carcinoma (Franke et al., 2005). Despite the unknown mechanisms of ethanol-mediated gastric ulcers, still, multiple pieces of evidence show that pro-inflammatory cytokines, oxidative damage, and cellular apoptosis play a vital role in its ethanol-induced progression (Al Batran et al., 2013; Luo et al., 2013). Various additional factors, such as the environment (cigarettes, alcohol, and infectious agents), chronic use of pain relievers such as nonsteroidal anti-inflammatory medicines, and incidents of stress, are among the leading causes of stomach ulcers (Tabiri et al., 2016). Changes in the stomach mucosal barrier, blood flow, and degenerative gastric secretion are all contributors to gastric ulcers in terms of major pathophysiology (Poonam et al., 2005). Due to disturbance in the mucosal blood flow, angiogenesis, and reduction of cell proliferation, ulcer healing in cigarette smokers is delayed (Zhang et al., 2012).

*Helicobacter pylori* (*H. pylori*) is a Gram-negative bacterium that can cause chronic gastritis, peptic ulcers, gastric adenocarcinoma, and mucosa-associated lymphoid tissue lymphoma in humans. The infection caused by this pathogen estimates about half of the world's population (Zamani et al., 2018). However, infection rates vary by region, with developing countries having a higher frequency than developed countries.

The proton pump in the stomach, gastric H^+^/K^+^-ATPase, is principally responsible for the acidification of the stomach contents. All stimulations of stomach acid production lead to the proton pump which is the common and final pathway. Blocking the activity of H^+^/K^+^-ATPase, which inhibits gastric acid output, is a popular clinical intervention for dyspepsia, peptic ulcer, and gastroesophageal reflux disease (Zhang et al., 2014). Ethanol causes changes in the cytokine equilibrium in the stomach mucosa, which causes inflammation (Almasaudi et al., 2016). Antioxidants seemed to have a protective role in gastric ulcers and carcinomas, whereas any imbalance in the activity of these oxidative stress enzymes usually results in improper free radical disposal, which results in the ulceration of gastric tissues (Tandon et al., 2004). An adequate number of anti-ulcer therapies are in practice, which has brought significant improvement in the ulcerative patient’s life.

Triple-based eradication therapy, which comprises a proton pump inhibitor (PPI) and two antimicrobial drugs, amoxicillin and clarithromycin, is thought to be the most effective treatment for *H. pylori*-induced ulcer eradication (Tulassay et al., 2008). Currently, commercially available worldwide PPIs are omeprazole, lansoprazole, and pantoprazole. PPIs are the most powerful stomach acid-lowering medicines in clinical usage because they irreversibly impair proton pump H^+^/K^+^-ATPase action. Proton pump inhibitors have been the treatment of choice for stomach acid hypersecretion (Richardson et al., 1998). Joint discomfort, irregular heartbeat, hemopoietic changes, gynecomastia, impotence, and systemic alkalosis are all common side effects of these medicines. Nowadays, more research is being conducted on the utilization of natural products in the development of medications with fewer adverse effects (Mahmoud and Ghffar, 2019).

The ethanol-induced gastric ulcer rodent model is considered a well-established model that reflects several features of human gastric injury and thus provides a means for testing unexplored test samples with anti-ulcer potential (Liu et al., 2012; Salga et al., 2012).

**FIGURE 1:**
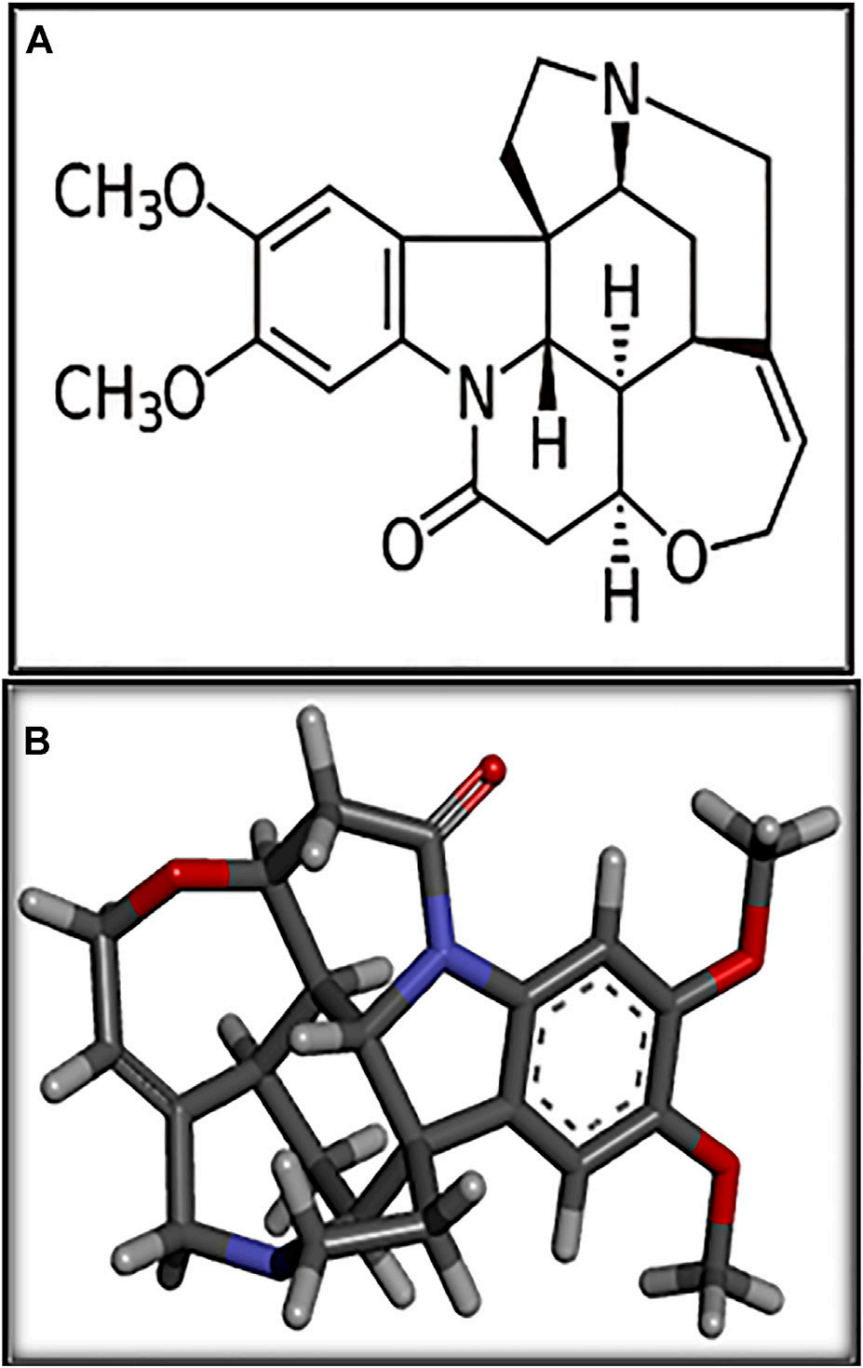
**(A, B)** represent 2D and 3D structures of brucine.

Brucine (Figure 1), one of the principal bioactive constituents isolated from the seeds of *Strychnos nux-vomica* L. (Loganiaceae), is a weak indole alkaloid. Maqianzi, also known as Nux-vomica, has a bitter flavor (Lu et al., 2020). Wide pharmacological activities of brucine have been reported by many basic and clinical researchers such as anti-tumor (Qin et al., 2012), anti-inflammatory and analgesic (Yin et al., 2003), and cardio-protective (Liu et al., 2021) activities. To date, to the best of our knowledge, no detailed study has been conducted that highlights the possible therapeutic potential of brucine in gastric ulcers. The current study has been conducted to evaluate the brucine anti-ulcer effect using molecular, *in silico*, *in vitro*, and *in vivo* techniques.

## Material and methods

### Chemicals

All standard chemicals used were purchased from verified sources. Brucine was purchased from Sigma CO, Merck. Normal saline, absolute ethanol, and chloroform were acquired from Sigma-Aldrich (St. Louis, MO, United States). Omeprazole and metronidazole were purchased from Barrett Hodgson and Sanofi Aventis, Pakistan. Abcam, United Kingdom, provided secondary antibodies. Elabscience, China, provided the rat NFB ELISA kit (catalog no. E-EL-R0674), rat TNF-α ELISA kit (catalog no. E-EL-ROO19), and rat PGE2 ELISA kit (catalog no. E-EL-R0034). All of the chemicals used in this experiment were of analytical quality.

### Animals

Sprague–Dawley rats weighing 150–200 g of either gender, purchased from the Riphah Institute of Pharmaceutical Sciences (RIPS) in Islamabad, were subjected to conduct this research work. The rats were kept in standard cages with a constant temperature of 22°C. All of the animals were fed a regular meal and had unlimited access to water. The experiments were performed by following the guidelines and principles of the Institute of Laboratory Animal Resources, Commission on Life Sciences University, National Research Council (1996). The study was approved by the Research and Ethics Committee of RIPS (Ref. No. REC/RIPS/2017/006).

### Computational study

The three-dimensional structure of a typical medication was obtained using a Biovia Discovery Studio Visualizer (DSV). The standard drug omeprazole was used. In gastric ulcer pathophysiology, human protein targets (3D structure) involved were selected and acquired from an online protein data bank, Research Collaboratory for Structural Bioinformatics (RCSB) PDB. The target proteins selected were H^+^/K^+^-ATPase (PDB ID: 5ylu), histaminergic (H_2_) (PDB ID: P25021), cyclooxygenase-1 (COX-1) (PDB ID: 6Y3C), tumor necrosis factor (TNF-α) (PDB ID: 1BKC), nuclear factor kappa B (NFκB) (PDB ID: 4Q3J), prostaglandin (PGE_2_) (PDB ID: 6AK3), cyclooxygenase-2 (COX-2) (PDB ID: 5IKQ), and muscarinic (M_1_) (PDB ID: 5CXV). The ligand and water molecules were removed from DSV, H-atoms (polar) were inserted, and the file was saved in PDB format. For molecular docking, the AutoDock tool-1.5.6 and PyRx-0.8 docking tools were used. The atomic contact energy (ACE) (Kcal/mol) value was calculated from the results. For post-dock analysis, the best pose with the lowest ACE value (kcal/mol) was chosen. 2D and 3D images were assessed to determine interactions between valine (VAL), tyrosine (TYR), tryptophan (TRP), threonine (THR), serine (SER), proline (PRO), phenylalanine (PHE), isoleucine (ILE), histidine (HIS), glycine (GLY), glutamine (GLN), glutamic acid (GLU), cysteine (CYS), and aspartic acid (CYS) residues and ligand residue (ALA).

### Anti-*H. pylori* activity

By the disc diffusion method, the antibacterial activity of the selected natural compound against *H. pylori* was evaluated. From the gastric biopsy of a patient with gastric ulcer, three strains of *H. pylori* were received under consent at the care endoscopy clinics and laboratories (Rawalpindi, Pakistan). Biopsies to be analyzed were placed in a modified Campy-Thio medium. Plates were kept at 37°C in a microaerophilic environment. Identification of isolates was carried out by morphology and using a urease test kit. Isolates were kept at 80°C in sterile McCartney bottles containing 0.2 g/L cysteine and 20% glycerol in brain heart fusion broth. Frozen clinical isolates were injected onto Mueller–Hinton agar plates for subsequent inoculation. Brucine with various concentrations of 0.5, 1, 2, 4, 8, 16, and 32 µg/disc was run on standard disks and then placed on a Mueller–Hinton agar plate. Following an incubation period of 3 to 5 days at 37°C, the zone of inhibition for each disk was measured. All tests were carried out in triplicate, and the antibacterial activity was calculated as the average inhibitory diameter (mm). Metronidazole was used as a positive control (Foroumadi et al., 2008).

### Ethanol-induced gastric ulcer

For gastric lesion induction, 24 h fasted rats were randomly assigned into five different groups (*n* = 5). Body weight saline (10 ml/kg) was given to group I as a negative control. Group 2 was treated with ethanol at a dose of 1 ml/100 g. Groups 3 and 4 were pretreated with brucine at doses of 1 and 3 mg/kg (p.o), respectively, and group 5 received omeprazole (20 mg/kg) as a standard drug it was used. After 1 hour of all treatments, absolute ethanol (1 ml/100 g) was orally administered to each rat except the animals of group 1, which served as a negative control. All rats were sacrificed by cervical dislocation after 1 h of ethanol administration. The stomachs of the participants were separated and cleaned with normal saline before the lesion index was computed by measuring each lesion in millimeters along its greater curvature. Each lesion’s covered surface area was measured and evaluated using the method provided by Qazi et al. (2017). The ulcer index was calculated as the mean ulcer score for each gastrointestinal lesion (US) where 0 indicates no ulcer, 1: US ≤ 0.5 mm^2^, 2: 0.5 < US ≤ 2.5 mm^2^, 3: 2.5 mm^2^ < US ≤ 5 mm^2^, 4: 5 mm^2^ < US ≤ 10 mm^2^, 5: 10 mm^2^ < US ≤ 15 mm^2^, 6: 15 mm^2^ < US ≤ 20 mm^2^, 7: 20 mm^2^ < US ≤ 25 mm^2^, 8: 25 mm^2^ < US ≤ 30 mm^2^, 9: 30 mm^2^ < US ≤ 35 mm^2^, and 10: US > 35 mm^2^. For each stomach, the sum of the length (mm) of all lesions was used as the ulcer index (UI). The percentage inhibition (% I) was calculated using

I(%)=(USc-USt)100/USc,

where USc = the ulcer surface area of control, and USt = the ulcer surface area of the test drug group.

### H^+^/K^+^-ATPase inhibitory activity

Using the calorimetric method, the H^+^/K^+^-ATPase inhibitory effect of brucine was analyzed at (3 mg/kg) using a commercially available calorimetric H^+^/K^+^-ATPase screening kit having catalog no: E-BC-K122-S; Elabscience, United States. The H^+^/K^+^-ATPase activity was assessed after ATP hydrolysis by monitoring inorganic phosphate levels. The homogenate of stomach tissue was centrifuged for 10–15 min at 3,500 rpm. As a result of the supernatant, spectrophotometrically at 660 nm, the inorganic phosphate release was determined. One ATPase activity unit has been described as one µ mole of inorganic phosphorus released by ATP hydrolysis through ATPase at 1 mg per hour of tissue protein. It was then expressed as µmol pi/mg prot/hour (Zhang et al., 2014).

### Antioxidant profile

The antioxidant activity of brucine was analyzed in the isolated tissues of the pretreated animals and compared with disease and positive control group tissues. The stomach tissues were homogenized and centrifuged at 1,500 rpm for 30 min to collect the supernatant. The obtained supernatant was estimated for glutathione (GSH), catalase, glutathione-S-transferase (GST), and lipid peroxidation (LPO). The oxidation of GSH and DTNP produced a yellow end product, which was used to determine their levels. Using a GSH microplate reader, the absorbance of 2-nitro-5-thiobenzoic acid was determined at 412 nm. The extinction coefficient of the product produced was used to calculate the GST activity, which was reported as mole/mg of CDNB conjugate/min/mg of protein in moles/mg of protein. The synthesis of the CDNB conjugate was used to determine the GST level, and the absorbance at 340 nm was measured. In the presence of catalase, the degradation of H_2_O_2_ was measured. A microplate reader was used to measure absorbance at 340 nm. The end product of LPO, malondialdehyde, was used to determine the level of LPO (MDA). A microplate reader was used to measure the absorbance at a wavelength of 532 nm, where quantitative measurement of LPO was expressed in TBARS nmoles/min/mg of protein (Irshad et al., 2021).

### Hematoxylin and eosin staining

On coated slides, in 100 percent pure xylene, tissue sections were deparaffinized and then rehydrated in 70 percent ethyl alcohol. The slides were immersed in hematoxylin for 10 min after being cleaned with distilled water. Then, in a glass jar, they were placed under running water for 10 min and treated with 1% HCl and 1% ammonia water. The slides were immersed in eosin solution for 5–10 min. After the required length of time had passed, the slides were cleaned in water and air–dried. In graded ethyl alcohol, the dried slides were dehydrated (70 percent, 95 percent, and 100 percent). The slides were cleaned with xylene and mounted with glass coverslips. The slides were photographed using an Olympus light microscope and analyzed by ImageJ, a computer-based application, with particular attention paid to the gastric cell size and shape, inflamed infiltrating cells, and vacuolation. The TIF images were arranged at the same threshold intensity for all groups and examined in GraphPad Prism.

### Immunohistochemistry

The immunohistochemical examination was carried out, as previously described by Gim et al. (2015). The slides were deparaffinized, then treated for antigen retrieval (enzymatic technique), and washed with PBS. For 10 min, 3 percent hydrogen peroxide was used to block the endogenous peroxidase in methanol (H_2_O_2_). The slides were incubated for a period of time in a solution containing 0.1 percent Triton X-100 and 5 percent normal goat serum. After being blocked, the slides were treated overnight with murine anti-TNF-α, p-NFkB, and anti-COX-2 antibodies (dilution 1:100, Santa Cruz Biotechnology). Slides were handled for incubation with the biotinylated secondary antibody (dilution 1:50), depending on the source of the primary antibody, and the serum was used the next morning after cleaning with 0.1 M PBS. The ABC Elite kit was used to incubate slides in a humidified room for 1 h after secondary antibody application (Santa Cruz Biotechnology). The slides were cleaned in 0.1 M PBS, dyed with DAB solution, rinsed with distilled water, dehydrated in a graded ethanol series, settled in xylene, and mounted. A light microscope was used to capture immunohistochemical TIF pictures. By modifying the backdrop of photos according to the threshold intensity and analyzing p-NFκB, COX-2, and TNF-α positive cells at the same threshold intensity for all groups, ImageJ software was utilized to quantitatively detect hyperactivated p-NFκB, COX-2, and TNF-α. The relative integrated density of the samples compared to saline is used to compute the intensity (Ansari et al., 2019).

### Enzyme-linked immunosorbent assay

TNF-α, PGE_2,_ and NFκB expressions were measured using the Rat TNF-α ELISA kit (catalog no: E-EL-R0019), Rat PGE_2_ ELISA kit (catalog no: E-EL-0034), and Rat NFκB ELISA kit (catalog no: E-EL-R0674), respectively, in accordance with the manufacturer’s guidelines (Elabscience). Using the Silent Crusher M (Heidolph) apparatus, the tissues were homogenized at 15,000 RPM; after centrifugation, the supernatant was collected (for 1 h at 1,350 g). TNF-α, PGE2, and p-NFκB concentrations were measured using an ELISA microplate reader. (Ansari et al., 2019).

### Western blot

Gastric tissues were homogenized after being lysed in a buffer. A bicinchoninic acid (BCA) protein test kit was used to assess the protein content. On a 12 percent sodium dodecyl sulfate-polyacrylamide gel, 30 g of protein homogenate was electrophoretically separated and transferred to a polyvinylidene fluoride membrane. Membranes were incubated overnight at 4°C with primary antibodies such as p-NFκB and TNF-α and then blocked for 1 h at room temperature with 5% bovine serum albumin. After being cleansed three times with Tris-buffered saline with 0.1 percent Tween 20, the membranes were further subjected to a 1:1,000 dilution of secondary antibodies, such as goat and anti-rabbit, for 90 min at room temperature. To see the immunoreactive bands, an enhanced Western blotting substrate kit was used. Densitometry was used to assess the measurement of protein expression by ImageJ software. (Irshad et al., 2021).

### Real-time polymerase chain reaction

The TRIzol technique was used to extract total ribonucleic acid (RNA) from gastric tissues, as directed by the manufacturer. Using a reverse transcriptase enzyme mix and a PCR thermocycler, the first strand of cDNA was generated from 1 to 2 g of total RNA. The mRNA expression of H^+^/K^+^-ATPase was normalized to the expression of β-actin as a house-keeping gene by the 2^-∆∆CT^ method. Primer sequences for H^+^/K^+^-ATPase and β-actin were CCC​GCG​AGT​ACA​ACC​TTC​T (forward) and CGTCATCCATGGCGAACT (reverse) and TAT​GAA​TTG​TAC​TCA​GTG​GA (forward) and TGGTCTGGTACTTCTGCT (reverse), respectively (Irshad et al., 2021).

### Statistical analysis

The data are presented as a mean with a standard error of the mean (SEM). GraphPad Prism 8 was used to apply statistical parameters such as one-way analysis of variance (ANOVA) with *post hoc* Tukey’s test. *p* < 0.05 was regarded as statistically significant.

## Results

### *In silico* analysis

In this study, brucine binds to a variety of protein receptors with varying affinities. In contrast to H^+^/K^+^-ATPase, H2, COX-1, TNF-α, NFκB, PGE2, COX-2, and M1, the values of atomic energy (kcal/mol) of ideal dock postures of brucine and reference drug, as well as residues implicated in H-bonding, pi-pi bonding, and other hydrophobic interactions, are shown. Supplementary Figures S1–S8 illustrate the 2D depiction of brucine interactions with common medications. Brucine had an E-value of −8.7 kcal/mol against the H^+^/K^+^-ATPase receptor and produced no H-bonds or hydrophobic contacts. Brucine had an E-value of -8.1 kcal/mol against H2 and produced two H-bonds, two bonds, and no hydrophobic interaction. Brucine has an E-value of −7.6 kcal/mol against COX-1, forming two H-bonds and no hydrophobic contacts. Brucine had an E-value of −8.11 kcal/mol against TNF-α and produced one H-bond, no bond, and no hydrophobic interaction. Brucine had an E-value of −8.58 kcal/mol against NFκB, forming two H-bonds and no hydrophobic interactions. Brucine had an E-value of −6.92 kcal/mol against PGE2 and produced no H-bonds, one bond, and no hydrophobic bonds. Brucine has an E-value of –2.99 kcal/mol against COX-2, forming one H-bond and no hydrophobic interactions. Brucine has an E-value of −8.74 kcal/mol against M1, forming one H-bond and no hydrophobic interactions. (Table 1).

**TABLE 1:** Binding energy (kcal/mol) and post-dock analysis of the best conformational pose of brucine with H^+^/K^+^-ATPase, histaminergic (H_2_) receptor, cyclooxygenase (COX-1), tumor necrosis factor (TNF-α), nuclear factor kappa B (NFκB), prostaglandin (PGE_2_), cyclooxygenase (COX-2), and muscarinic (M_1_) receptor.

Brucine						Standard drug				
Target protein	PDB ID	E-value (Kcal/mol)	H-bond	H-bonding residue	Binding residue forming other hydrophobic interactions	Drug name	E-value (Kcal/mol)	H-bond	H-bonding residue	Binding residue forming other hydrophobic interactions
H^+^/K^+^-ATPase	5YLU	−8.7	—	—	ASN138	Omeprazole	−8.2	2	CYS813	ILE816
					ARG328					LEU141
					VAL331					LEU796
					GLN127					TYR799
					THR135					ALA335
					ASP137					ALA339
H_2_	H2P2501	−8.1	2	ASN292TYR202	THR53	Ranitidine	−8.5	—	—	VAL113
					ILE112					TRP387
					ILE57					TYR381
					TYR288					ALA196
COX-1	6Y3C	−7.6	2	HIS90	GLY354	Aspirin	−6.9	1	LYS231	ARG116
				HIS95	ASN515					ASN54
					GLN192					ASN292
					SER353					TYR288
					SER516					TYR202
TNF-α	1BKC	−8.11	1	PRO437	HIS415	Aspirin	−6.2	3	SER121	THR118
					ILE438				GLN372	ASN122
					ALA439				LYS532	
					HIS405					
					LEU348					
NFκB	4Q3J	−8.58	2	ASN240	TYR227	Curcumin	8.42	4	GLN372	TYR374
				ARG23	GLU207				LYS532	PRO543
					GLU179				SER121	ALA544
					GLY180				ILE124	SER126
					GLU184					HIS122
PGE_2_	6AK3	−6.92	-	-	TRP344	Dinoprostone	8.24	5	ARG333	PHE140
									THR206	
									TYR114	
									SER336	
									THR107	
COX-2	5IKQ	−2.99	1	SER127	ASP126	Meclofenamate	7.13	4	GLU184	PRO147
					ALA543				CYS149	ILE148
					PRO542				ARG237	LEU236
					SER541				ASN240	
M_1_	5CXV	−8.74	1	ILE180	TYR85	Phenoxy benzamine	−5.20	4	GLY349	THR347
					TYR82				HIS405	LEU348
					TRP400				HIS409	GLU406

Standard inhibitors or activators of pathways are omeprazole, phenoxy benzamine, ranitidine, aspirin, meclofenamate, dinoprostone, and curcumin. Amino acids are arginine (ARG), isoleucine (ILE), asparagine (ASN), tyrosine (TYR), histidine (HIS), threonine (THR), glutamic acid (GLU), proline (PRO), phenylalanine (PHE), valine (VAL), glycine (GLY), tryptophan (TRP), leucine (LEU), cystine (CYS), aspartic acid (ASP), alanine (ALA), and serine (SER).

### Anti-*H. pylori* effect

The minimum inhibitory concentration (MIC) and zone of inhibition of three different strains of *H. pylori* against brucine were measured. Three strains used were strain (A) J63 (cag A-), strain (B) j196 (cag A-), and strain (C) j107 (cag A+). Different concentrations of brucine were used against strain (A) that are 0.5, 1, 2, 8, 16, and 32 µg/disc, and zones of inhibition were calculated as 1, 1.33, 2, 4, 8, 10.66, and 14 mm, respectively, and its MIC is 16 μg/ml. The same concentration of brucine was used for strain (B), and the inhibition values are 1.33, 1.66, 2, 4.66, 7.33, 10, and 13 mm, respectively, and its MIC is 18 μg/ml. For strain (C), same concentrations of brucine were used, and the inhibitory values are 1.66, 2, 2.33, 4.33, 8.66, 11.66, 14, and 16 mm, respectively, and its MIC is 16 μg/ml, compared with the standard metronidazole, respectively (Table 2).

**TABLE 2:** Antibacterial effect of brucine against three clinical strains of *H. pylori* using the disk diffusion method.

Sample	Zone of inhibition (mm)							
	0.5 µg/disk	1 µg/disk	2 µg/disk	4 µg/disk	8 µg/disk	16 µg/disk	32 µg/disk	MIC_50_ (µg/ml)
**Strain J63 (cagA-)**								
Brucine	1 ± 0	1.33 ± 0.33	2 ± 0.57	4 ± act0.57	8 ± 0.57	10.66 ± 0.33	14 ± 0.57	16
Metronidazole	3.66 ± 0.33	4.66 ± 0.33	5.33 ± 0.66	7 ± 0.57	10.33 ± 1.20	14.66 ± 0.88	22 ± 1.15	4
**Strain J196 (cagA-)**								
Brucine	1.33 ± 0.33	1.66 ± 0.66	2 ± 0.57	4.66 ± 0.33	7.33 ± 0.33	10 ± 0.57	13 ± 0.57	18
Metronidazole	4 ± 0.57	5 ± 0.57	5 ± 0.57	7.33 ± 0.88	10.3 ± 1.20	15 ± 1.00	20.6 ± 1.33	6
**Strain J107 (cagA+)**								
Brucine	1.66 ± 0.33	2 ± 0.57	2.33 ± 0.33	4.33 ± 0.88	8.66 ± 0.33	11.66 ± 0.88	14 ± 0.57	16
Metronidazole	4 ± 0.57	4.66 ± 0.33	5.66 ± 0.88	8 ± 1.15	11.33 ± 0.66	15.6 ± 0.33	22.6 ± 0.66	4

### Effect on ethanol-induced ulcer

**FIGURE 2:**
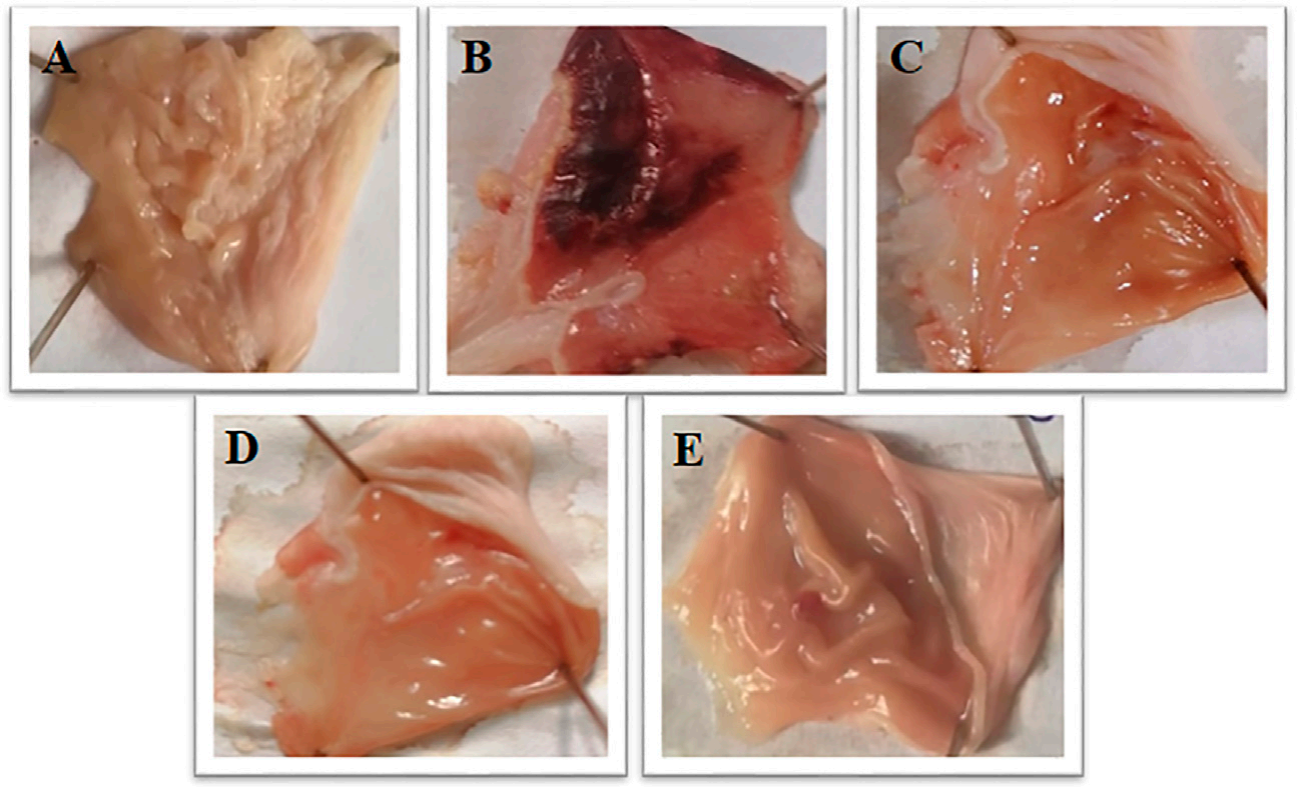
Gross appearance of gastric mucosa in rats: **(A)** pretreated with saline, (10 mL/kg). **(B)** Severe injuries are seen, extensive visible hemorrhagic necrosis of gastric mucosa was produced by absolute ethanol (1 mL/100g), **(C, D)** pretreated with brucine at doses of 1 and 3 mg/Kg, and **(E)** pretreated with omeprazole 20 mg/Kg.

Brucine at 1 and 3 mg/kg exhibited an anti-ulcer effect. Brucine shows an 80% protective effect at 1 and 3 mg/kg doses with ulcer indexes of 2 ± 0.93 and 2 ± 0.31, respectively. Omeprazole (20 mg/kg) exhibited a 90% inhibitory effect with the ulcer index 1 ± 0.13 as compared to the ethanol group (Table 3). The stomach mucosa of rats was observed under a microscope (Figure 2).

**TABLE 3:** Protective effect of brucine and omeprazole against ethanol-induced gastric ulcer in rats.

Treatment (mg/kg)	Ulcer index (UI)	% Inhibition
Saline (10 ml/kg)	0 ± 0	_
Ethanol (1 mL/100 g)	10 ± 0.00^###^	0
Brucine (1 mg/kg) + ethanol (1 mL/100 g)	2 ± 0.93^∗∗∗^	80
Brucine (3 mg/kg) + ethanol (1 mL/100 g)	2 ± 0.31^∗∗∗^	80
Omeprazole (20 mg/kg) + ethanol (1 mL/100 g)	1 ± 0.13^∗∗∗^	90

^###^ *p* < 0.001 compared to the control saline group; ^∗∗∗^ *p* < 0.001 vs. the ethanol group. One-way analysis of variance followed by *post hoc* Tukey’s test, ± SEM (*n* = 5).

### H^+^/K^+^-ATPase inhibition

**FIGURE 3:**
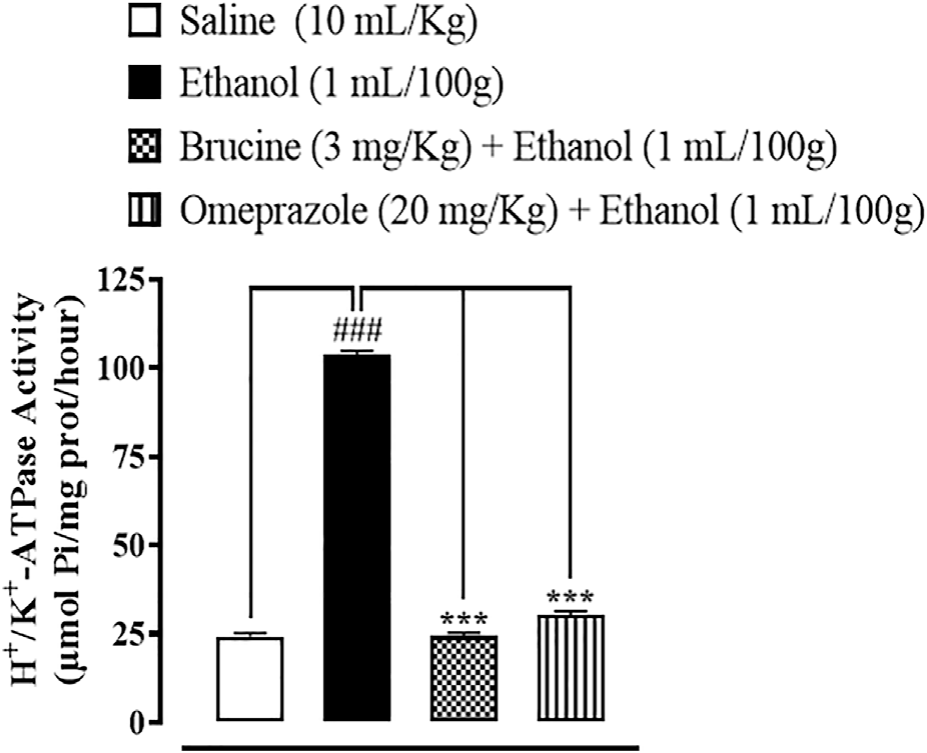
Inhibitory effect of brucine and omeprazole against H+/K+-ATPase in ethanol-induced ulcer rat gastric tissues. Values expressed as mean ± SEM (*n* = 5). One-way ANOVA with *post hoc* Tukey’s test was applied. ^###^p < 0.001 vs. saline group; ∗∗∗*p* < 0.001 vs. ethanol group.

In the ethanol-treated group, the H^+^/K^+^-ATPase activity was increased by 103.75 µmol Pi/mg prot/h, while brucine (3 mg/kg) and omeprazole (20 mg/kg) reduced H^+^/K^+^-ATPase by 24.34 µmol Pi/mg prot/h and 30.29 µmol Pi/mg prot/h, respectively (Figure 3).

### Effect on oxidative stress markers

**FIGURE 4:**
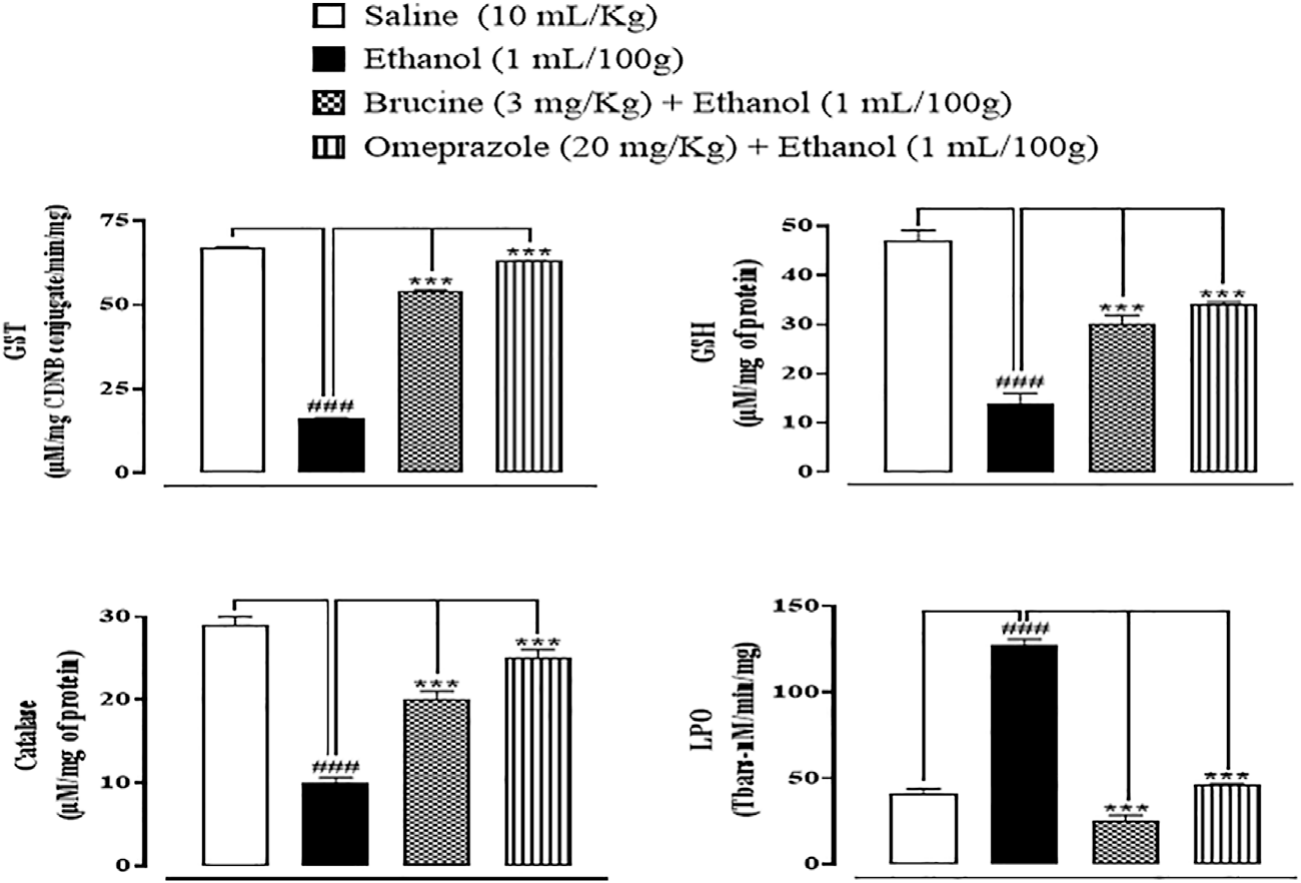
Effect of brucine and omeprazole against glutathione sulfotransferases (GST), reduced glutathione (GSH), catalase, and lipid peroxidase (LPO) in ethanol-induced ulcer rat gastric tissues. Data presented as mean ± SEM (*n* = 5). One-way ANOVA with *post hoc* Tukey’s test. ^###^ *p <* 0.001 vs. saline group; ^*∗∗∗*^ *p <* 0.001 vs. ethanol group.

In ethanol-induced ulcer stomach tissues, GST, GSH, and catalase levels were decreased, but LPO levels were increased. Brucine (3 mg/kg) and omeprazole (20 mg/kg)-treated groups significantly reduced LPO while restoring GST, GSH, and catalase (Figure 4).

### Histopathological examination

**FIGURE 5:**
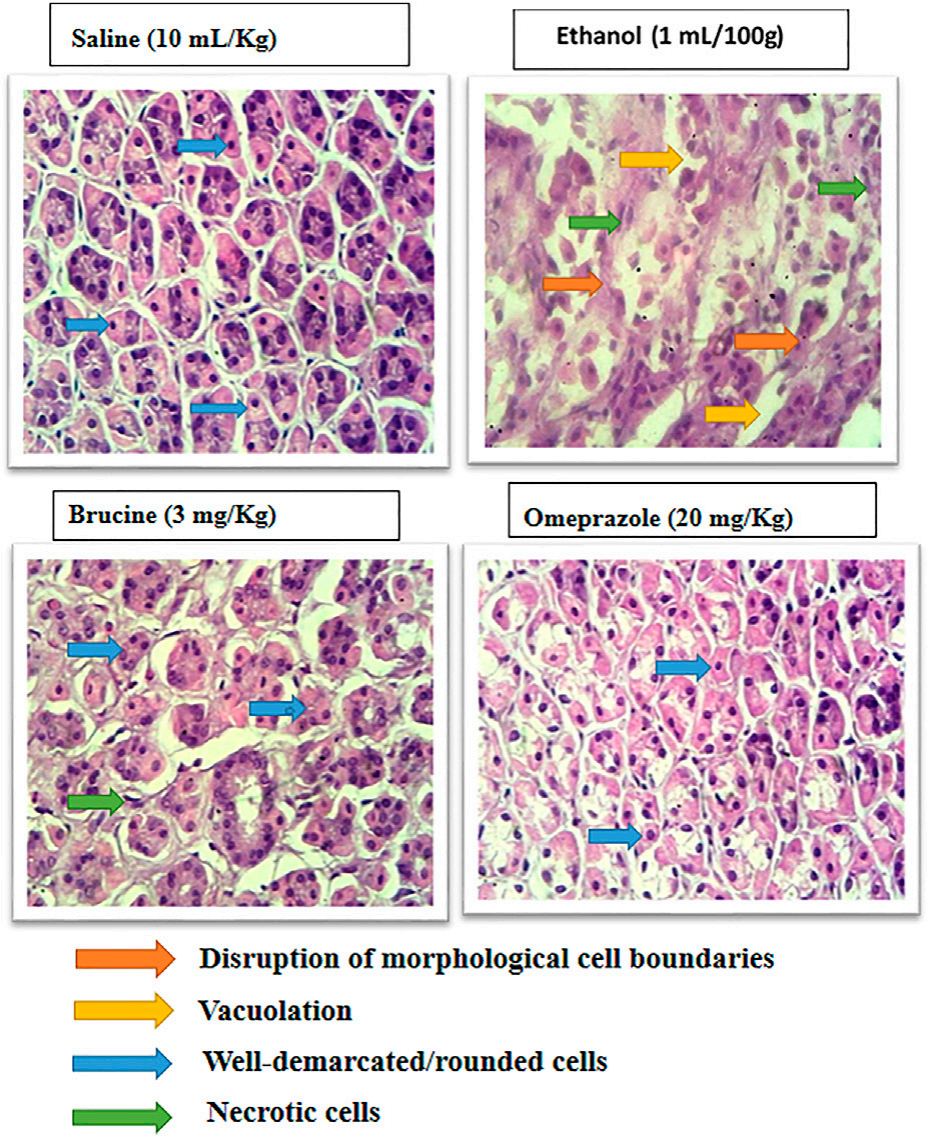
Histopathological slides represent effect of brucine and omeprazole in ethanol- induced ulcer rats gastric tissues. Saline group showing normal histological features. Ethanol ulcer group showing marked histopathological deformities by loss of stomach architecture, vacuolization and cloudy swelling. Brucine and omeprazole treatment groups showing near normal architecture with mild to moderate deformities.

Normal stomach tissues were seen in the saline (10 ml/kg) group, having architecture without any pathological changes. Ethanol (1 ml/100 g)-ulcerated rat tissues exhibited severe gastric tissues, damage with vacuolation, and disruption of morphological cell boundaries. The gastric tissues of mice treated with brucine (3 mg/kg) and omeprazole (20 mg/kg) demonstrated the regeneration and repair of gastric cells with minimal degradation (Figure 5).

### IHC analysis

**FIGURE 6:**
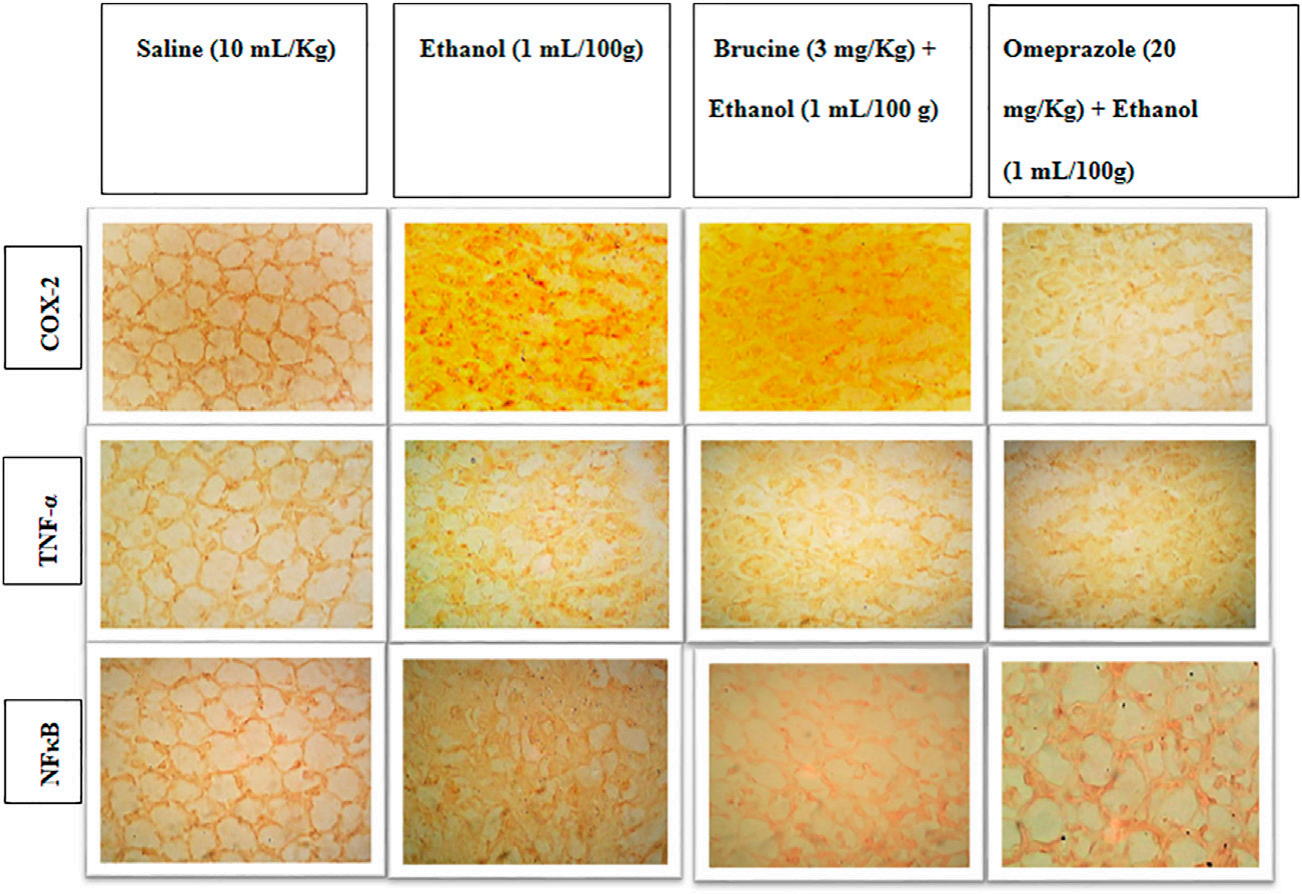
Slides represent the effect of brucine and omeprazole against the expressions of cyclooxygenase (COX-2), nuclear factor kappa B (p-NFκB), and tumor necrosis factor alpha (TNF-α) in ethanol-induced ulcer rat gastric tissues, using the immunohistochemical technique.

**FIGURE 7:**
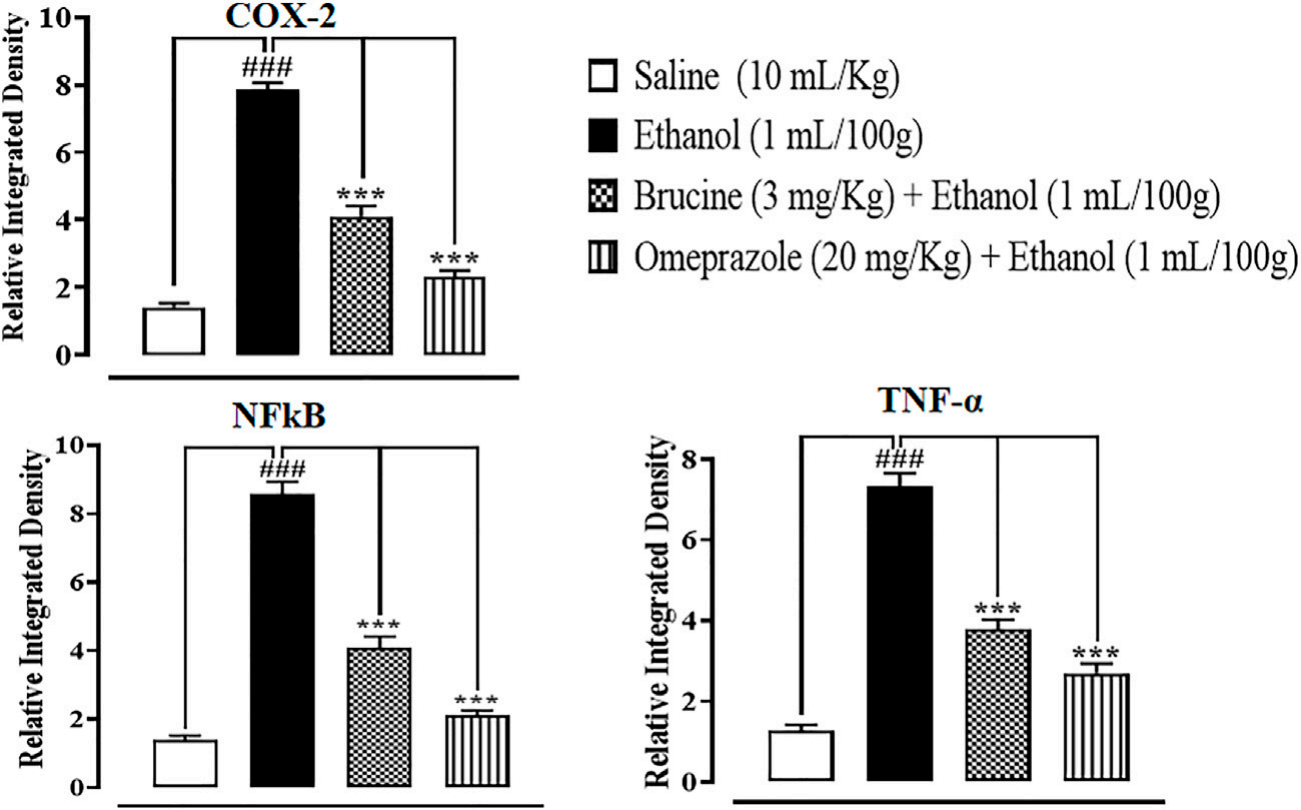
Effects of brucine and omeprazole against cyclooxygenase (COX-2), nuclear factor kappa B (p-NFκB), and tumor necrosis factor alpha (TNF-α) in ethanol-induced ulcer rat gastric tissues, using the immunohistochemical technique. Values expressed as mean ± SEM (*n* = 5). Data were analyzed by one-way ANOVA, followed by *post hoc* Tukey’s test. ^###^ *p < 0.001* vs. saline group. ^*∗∗∗*^ *p <* 0.001 vs. ethanol group.

The results of IHC on stomach tissues revealed that the ethanol (1 ml/100 g)-treated group markedly upregulated inflammatory markers COX-2, p-NFκB, and TNF-α expression. Vacuolation, necrotic cells, and disruption of morphological cell boundaries were found in the disease group. Brucine (3 mg/kg) and omeprazole (20 mg/kg) groups downregulated COX-2, p-NFκB, and TNF-α expressions (Figures 6, 7).

### Effect on inflammatory markers

**FIGURE 8:**
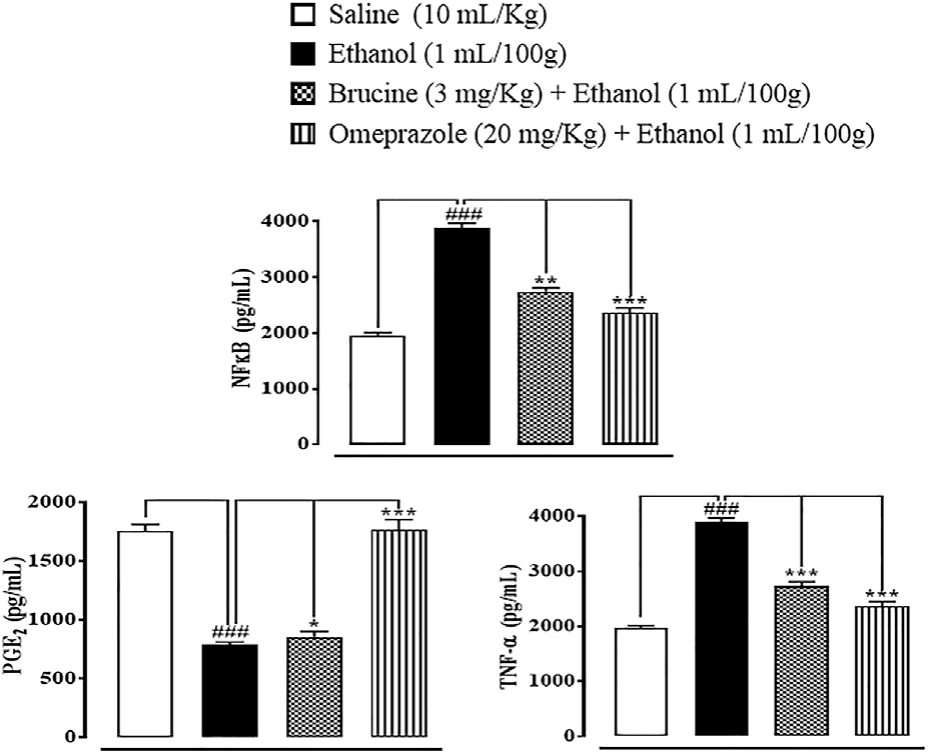
Effects of brucine and omeprazole against nuclear factor kappa B (p-NFκB), prostaglandin (PGE_2_), and tumor necrosis factor (TNF-α) levels in ethanol-induced ulcer rat gastric tissues, using the enzyme-linked immunosorbent assay technique. Values expressed as mean ± SEM (*n* = 5). One-way ANOVA with *post hoc* Tukey’s test. ^###^ *p <* 0.001 vs. saline group. *p < 0.05, ∗∗*p <* 0.01, and ∗∗∗*p <* 0.001 vs. ethanol group.

In gastric tissues of the saline (10 ml/kg)-treated group, p-NFκB, TNF-α, and PGE_2_ levels were 1960 ± 50, 1980 ± 30, and 1755 ± 55 pg/ml, respectively. In the ethanol (1 ml/100 g)-treated group, p-NFκB and TNF-α levels increased to 3,888 ± 88 and 3,896 ± 75 pg/ml, and the PGE_2_ level decreased to 785 ± 25 pg/ml, respectively. In the brucine (3 mg/kg)-treated group, p-NFĸB and TNF-α levels decreased to 2,740 ± 70 and 2,730 ± 80 pg/ml, and the PGE_2_ level increased to 850 ± 50 pg/ml, respectively. In the omeprazole (20 mg/kg)-treated group, p-NFκB and TNF-α levels decreased to 2,365 ± 85 and 2,365 ± 85 pg/ml, and the PGE_2_ level increased to 1765 ± 85 pg/ml, respectively (Figure 8).

### Western blot findings

**FIGURE 9:**
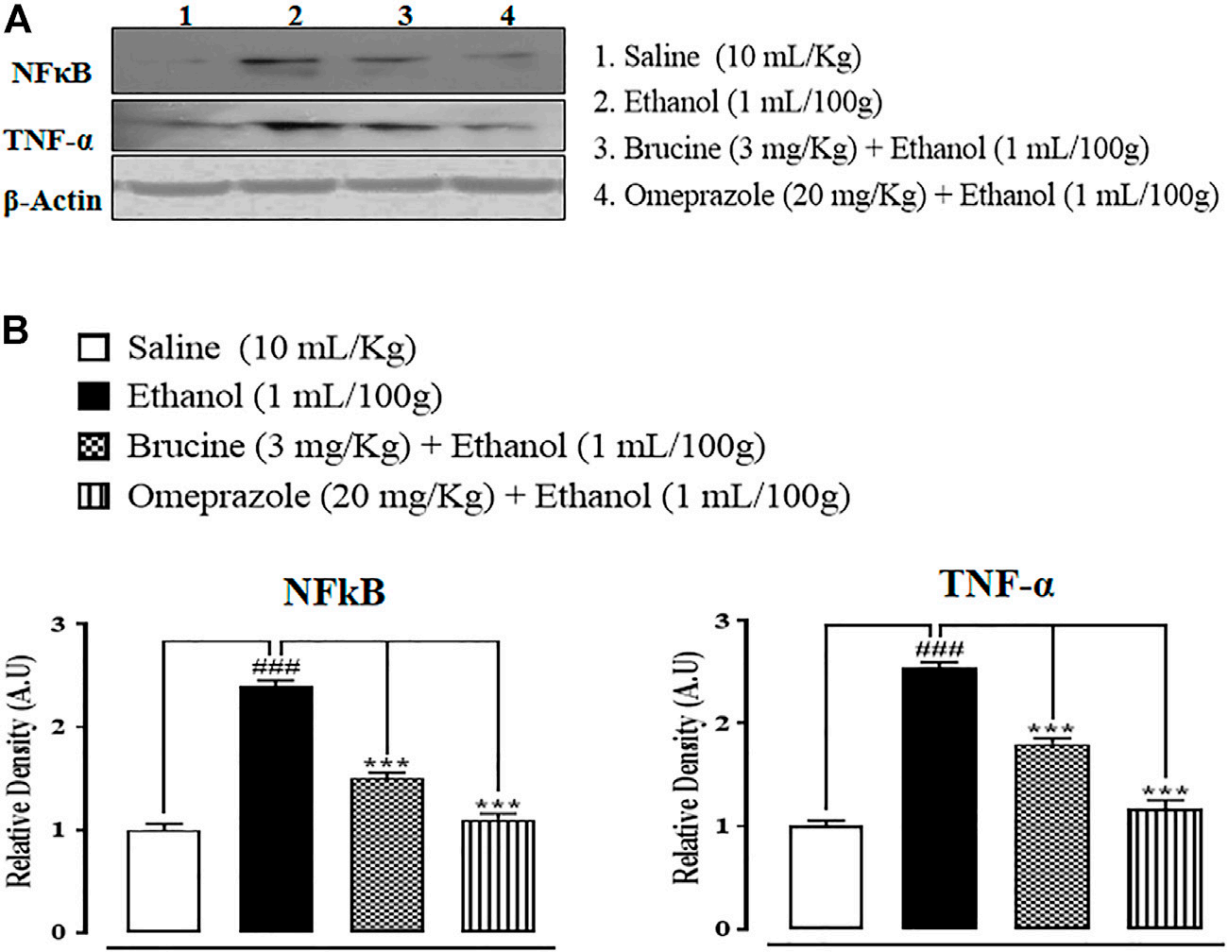
Bands **(A)** and graphical **(B)** representation of effects of brucine and omeprazole against phosphorylated nuclear factor kappa B (p-NFκB) and tumor necrosis factor (TNF-α) expressions in ethanol-induced ulcer rat gastric tissues, using the Western blot technique. Values expressed as mean ± SEM (*n* = 5). One-way ANOVA with post hoc Tukey’s test. ^###^ *p* < 0.001 vs. saline group. ***p < 0.001 vs. ethanol group.

In the ethanol (1 ml/100 g) group, p-NFκB and TNF-α expressions in the gastric region were increased. In brucine (3 mg/kg)- and omeprazole (20 mg/kg)-treated groups, TNF-α and NFκB levels were suppressed (Figure 9).

### Quantification of the mRNA level

**FIGURE 10:**
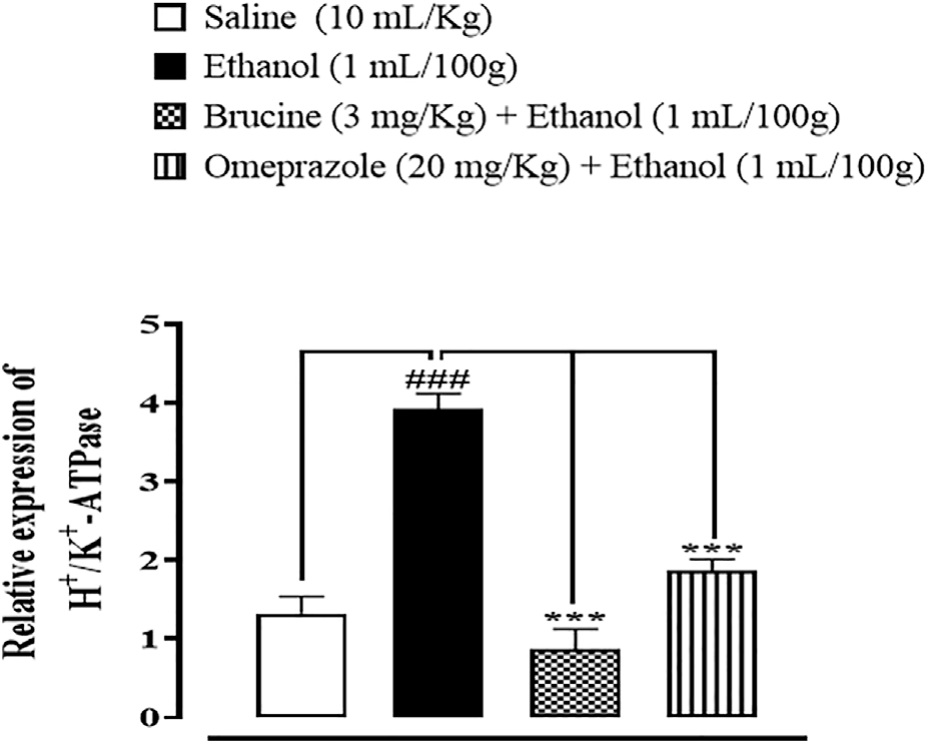
Effect of brucine and omeprazole against H^+^/K^+^-ATPase mRNA expression in ethanol-induced ulcer-treated rat gastric tissues, using the reverse transcription–polymerase chain reaction (RT-PCR) technique. One-way ANOVA followed by *post hoc* Tukey’s test. Values expressed as mean ± SEM (*n* = 5). ^###^ *p* < 0.001 vs. saline group; ∗∗∗*p <* 0.001 vs. ethanol group.

RT-PCR determined the fold expression of H^+^/K^+^-ATPase in ethanol-induced gastric ulcer. In the ethanol-treated group, the expression of H^+^/K^+^-ATPase mRNA levels increased. Brucine (3 mg/kg) and omeprazole (20 mg/kg) decreased H^+^/K^+^-ATPase mRNA levels (Figure 10).

## Discussion

This research work was conducted to explore the protective actions of brucine against ethanol-induced gastric ulcers in Sprague–Dawley rats. Ethanol is known for its notorious gastric ulceration by directly enhancing the disruption of mucosal cellular membranes, dehydration, and cytotoxicities, followed by the propagation of inflammatory cytokines, oxidative damage, and apoptosis (Park et al., 2008). The currently used model of ethanol-induced gastric ulcer is a well-established rodent model commonly implicated for preclinical evaluation of new drug molecules having anti-ulcer potential since ethanol has been regarded as a leading cause of gastric injuries in humans (Augusto et al., 2007). Drug discovery and development is incomplete without the evaluation of the structure of a compound. The majority of all potential drug molecules act by binding to specific protein targets, but the physical process by which these molecules and/or ligands bind to proteins remains difficult to explore. For this purpose, computational studies were carried out where the ligand was docked with its respective target using compound and protein databases (Nayarisseri, 2020). A docking tool was used for analysis of the ligands’ binding affinity to their respective protein targets (Jakhar et al., 2020). Brucine compound showed enormous significant binding affinity against various protein targets, and the ligand affinity order was found as TNF-α > M_1_ > H^+^/K^+^-ATPase > NFκB > H_2_ > COX-1 > PGE_2_ > COX-2. This study displayed results of all ligands compared with standard drugs, obtained from the PubChem database and RCSB.

In the present study, the ethanol-induced gastric ulcer model was used for *in vivo* experimentation (Li et al., 2014). In an *in vivo* study, pretreatment of rats with brucine at both doses significantly reduced the ulcer index relative to the disease group. In comparison to the usual treatment, ulcerated animals pretreated with brucine demonstrated a greater reduction in the ulcer index as compared to omeprazole (Abebaw et al., 2017). In human and experimental animals, oxygen-derived free radicals have been implicated in the etiology of a wide range of clinical diseases and stomach injuries, resulting in gastrointestinal ulcers (Yeo et al., 2018).

In the *in vitro* conformational analysis, *H. pylori* is the main risk factor for gastric ulcer disease (Graham, 2014). Brucine possesses antibacterial activity as it inhibits *H. pylori* bacterium which is mainly responsible for gastric ulceration (Foroumadi et al., 2008). Anti-*H. pylori* activity was examined through the zone of inhibition and minimum inhibitory concentration. Brucine showed an anti-*H. pylori* effect through the zone of inhibition and MIC against three different clinical strains.

H^+^/K^+^-ATPase has a key role in gastric ulcer. The proton pump in the stomach and the enzyme that is responsible for acidification of the stomach contents are known as gastric H^+^/K^+^-ATPase (Zhang et al., 2014). In gastric acid secretion, the proton pump is the most prevalent and important stimulation mechanism. Blocking the activity of H^+^/K^+^-ATPase, which inhibits gastric acid output, is a popular clinical intervention for dyspepsia, peptic ulcer, and gastroesophageal reflux disease. Brucine showed effective results in the inhibition of the H^+^/K^+^-ATPase enzyme (Zhang et al., 2013).

Considerable evidence shows that inflammation is followed by oxidative stress in gastric ulcer (Seifi et al., 2010). According to the molecular study, an increase in oxidative stress parameters is connected to lower levels of GSH, GST, catalase, and a higher level of LPO. These findings are in line with earlier research that suggests ulcerated animals’ tissue antioxidant defenses are compromised (Fu et al., 2018). Under oxidative stress, oxidative stress markers could represent free radical generation and defense against ROS creation (McGarry et al., 2018). In keeping with earlier observations that brucine has significant antioxidant activity, brucine prevents by boosting GSH, GST, and catalase while reducing LPO levels (Saraswati et al., 2013).

Brucine protected gastric cells by inhibiting inflammatory markers such as TNF-α, p-NFκB, and COX-2 when examined for cytokine levels by H&E staining and IHC, whereas these markers were significantly higher in the ethanol group. TNF-α, COX-2, and p-NFκB are prototypic pro-inflammatory cytokines because of their important role in initiating the cascade of cytokine activation and growth factors in the inflammatory response (Ansari et al., 2019).

For further confirmation, ELISA was carried out to quantify the inflammatory markers of TNF-α, p-NFκB, and PGE_2_. These inflammatory markers were significantly raised in ulcerative tissues (Zhou et al., 2016), while brucine significantly reduced these inflammatory markers, as shown in our current findings.

Western blot findings provide evidence that brucine has anti-inflammatory effects through reduced expressions of p-NFκB and TNF-α. Both proteins are involved in the recruitment of inflammatory mediators. Western blot results show increased expressions in the ethanol group of rats compared to the saline group, while brucine and omeprazole reduced these expressions significantly (He et al., 2018). Similar types of protective effects and suppression of inflammatory cytokines have been documented in earlier conducted studies on diosmine against an ethanol-induced gastric ulcer rat model (Arab et al., 2015).

The RT-PCR technique provides further evidence and confirmation of targeted H^+^/K^+^-ATPase. The amounts of H^+^/K^+^-ATPase mRNA were measured in the saline, ethanol, treatment, and omeprazole groups. Results showed that H^+^/K^+^-ATPase mRNA levels increased in the ethanol group as compared to the saline group, while treatment with brucine and omeprazole significantly reduced H^+^/K^+^-ATPase mRNA levels in the treatment group as compared to the ethanol group (Wang et al., 2017). It was confirmed from different experimental studies that brucine has a gastro-protective effect, mediated *via* anti-*H. pylori*, H^+^/K^+^-ATPase inhibition, and antioxidant and anti-inflammatory pathways.

## Conclusion

The current study highlights pieces of evidence for the protective effects of the brucine ethanol-mediated gastric injury rat model. Brucine possesses binding energy values ranging from F02D2.99 to F02D8.11 kcal/mol against selected targets. Brucine exhibits an anti-ulcer effect, mediated *via* anti-*H. pylori*, H^+^/K^+^-ATPase inhibition, and antioxidant and anti-inflammatory pathways. Thus, the current findings are a reflection of the potential benefits of brucine as an effective and safer candidate for the management of gastric injuries; however, we recommend further studies to investigate in detail the potential efficacy and safety of brucine in the clinical setting as an adjunct approach to address gastric injury-related health issues.

## Data Availability

The original contributions presented in the study are included in the article/Supplementary Material; further inquiries can be directed to the corresponding author.
