# Supplementary figures and images for: Pharmacological investigation of brucine anti-ulcer potential
Source: Front Pharmacol. 2022 Aug 17;13:886433. doi: 10.3389/fphar.2022.886433 (PMC9429807; doi:10.3389/fphar.2022.886433)

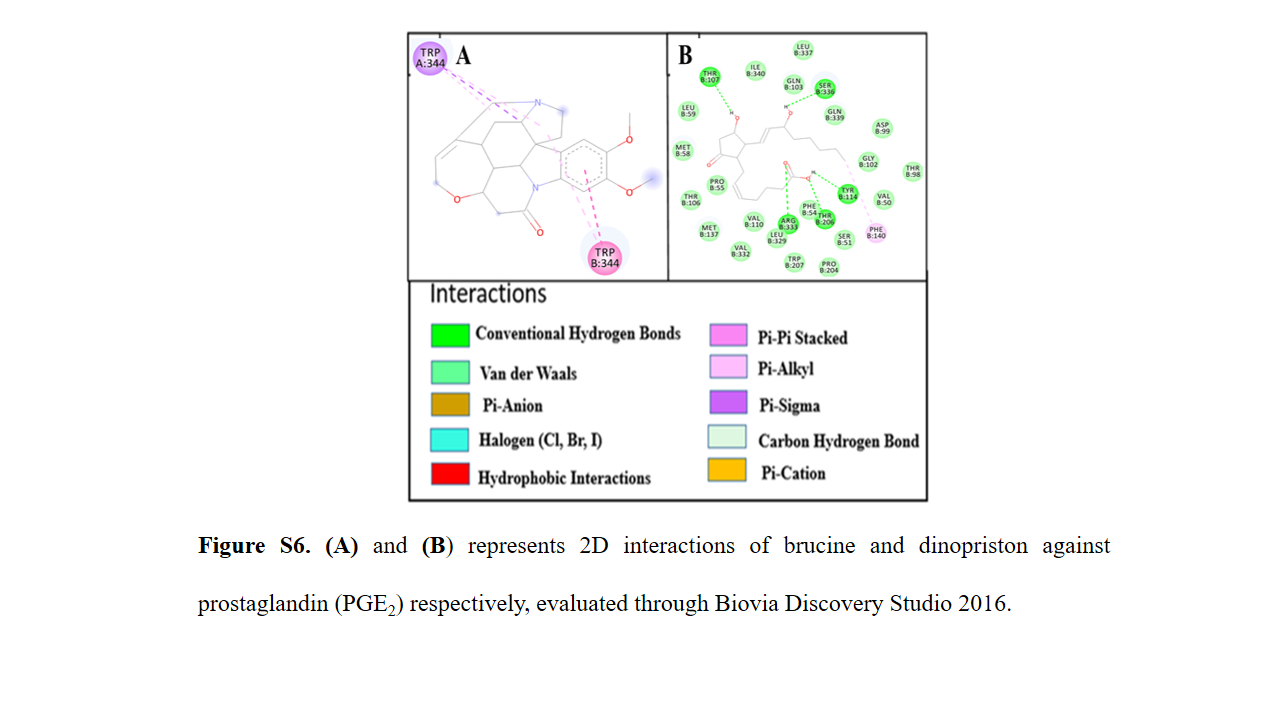

Supplement: Supplementary file 2 [file Image6.TIF]

**
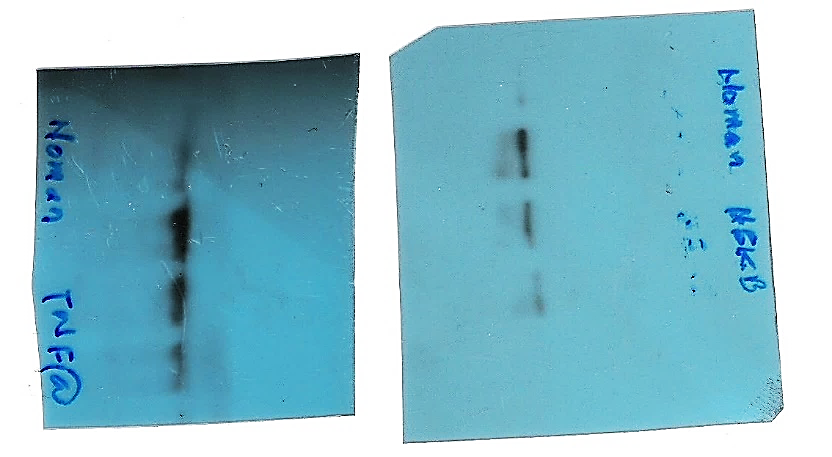

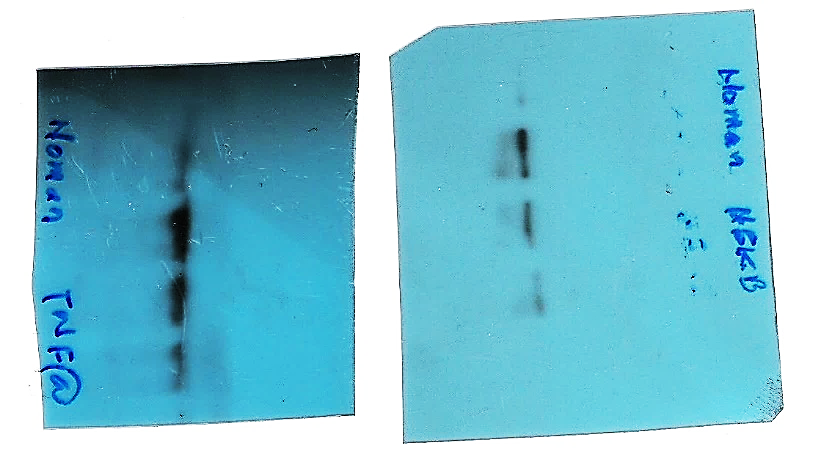
Uncropped data of Western Blot**

**
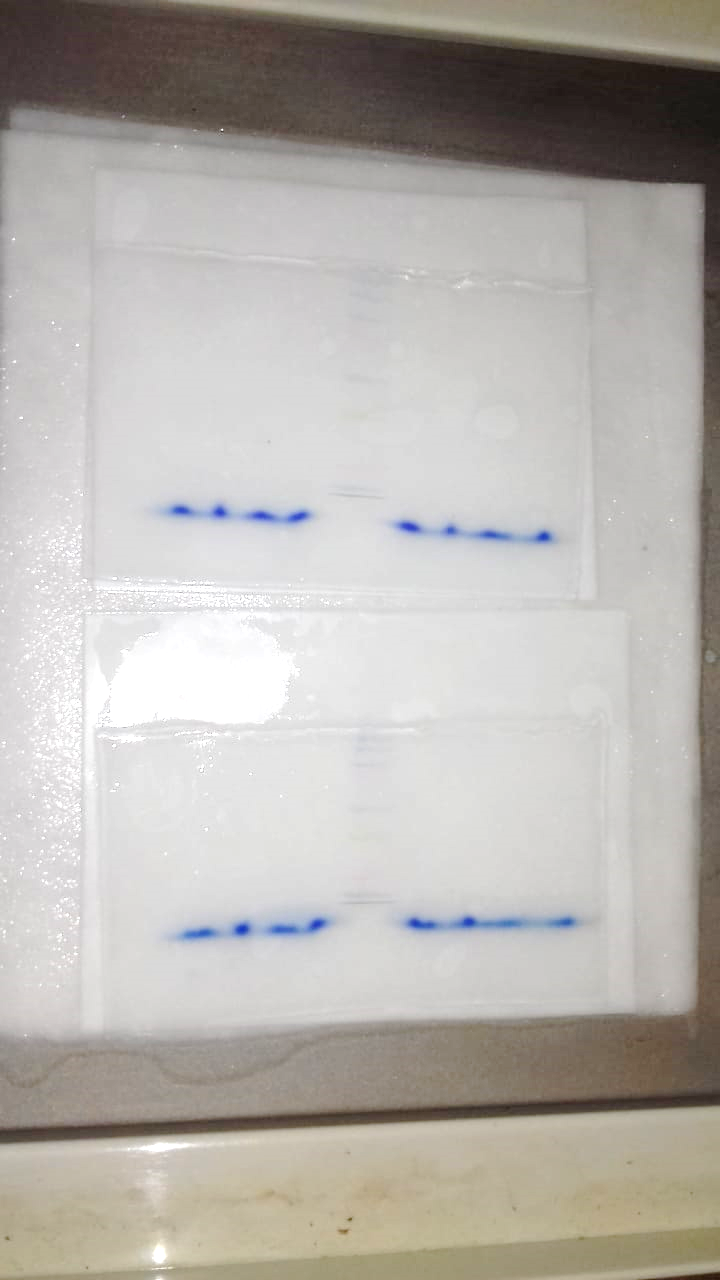
**

Supplement: Supplementary file 3 [file Table1.DOCX]

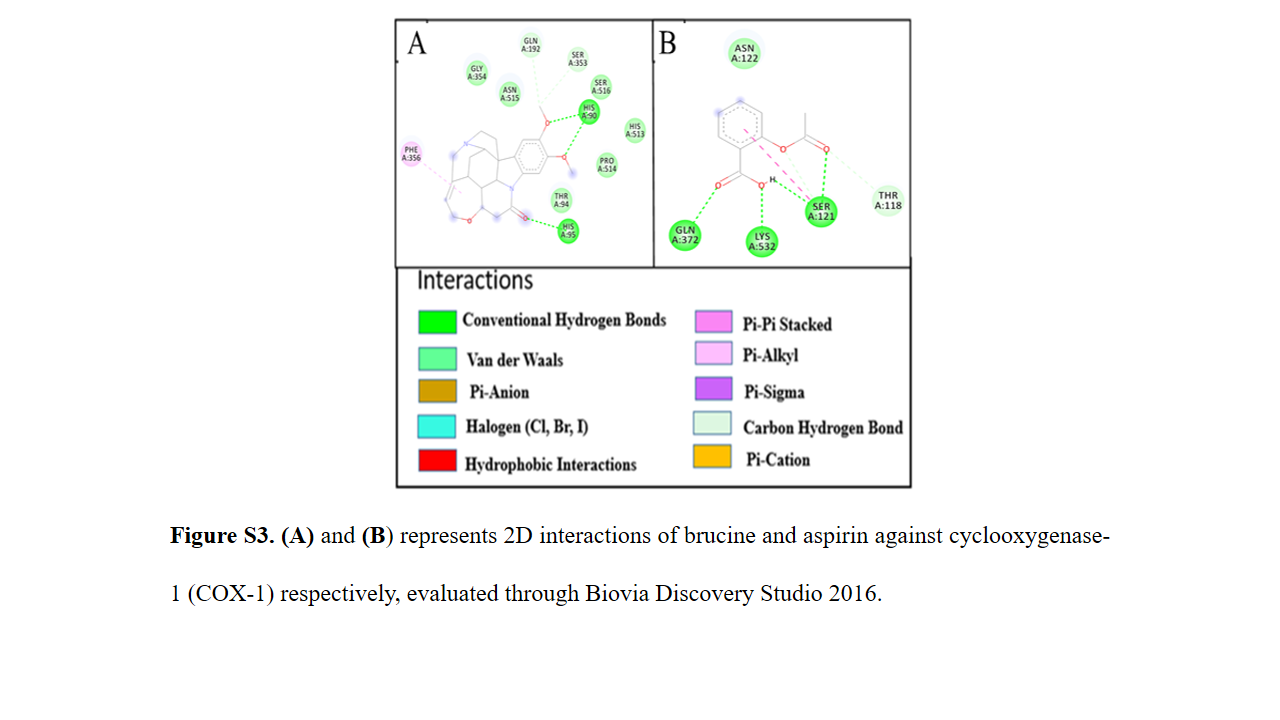

Supplement: Supplementary file 4 [file Image3.TIF]

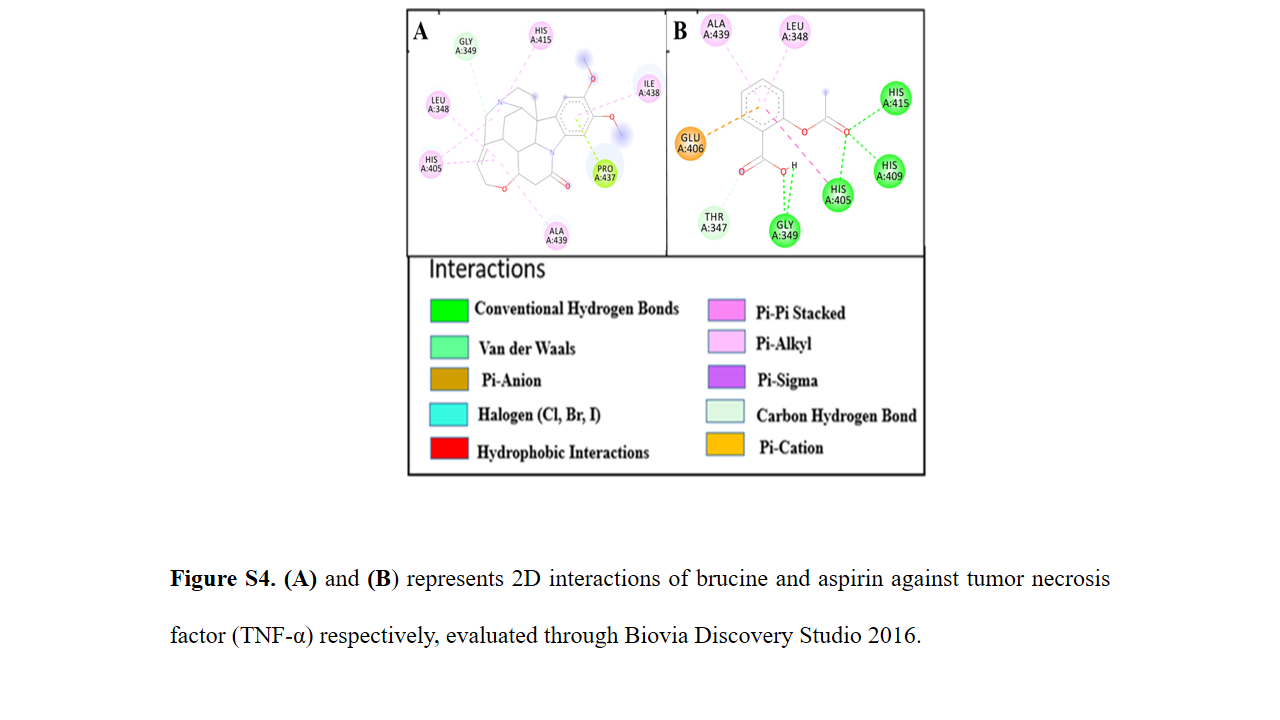

Supplement: Supplementary file 5 [file Image4.TIF]

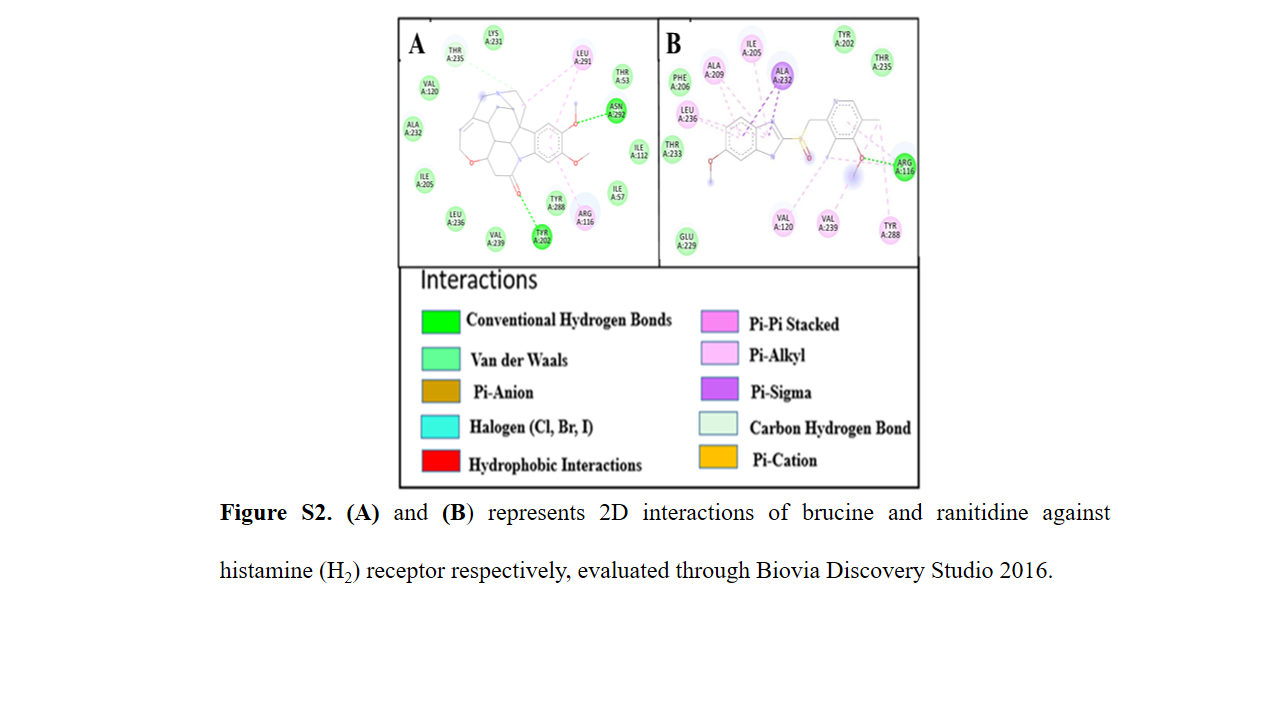

Supplement: Supplementary file 6 [file Image2.TIF]

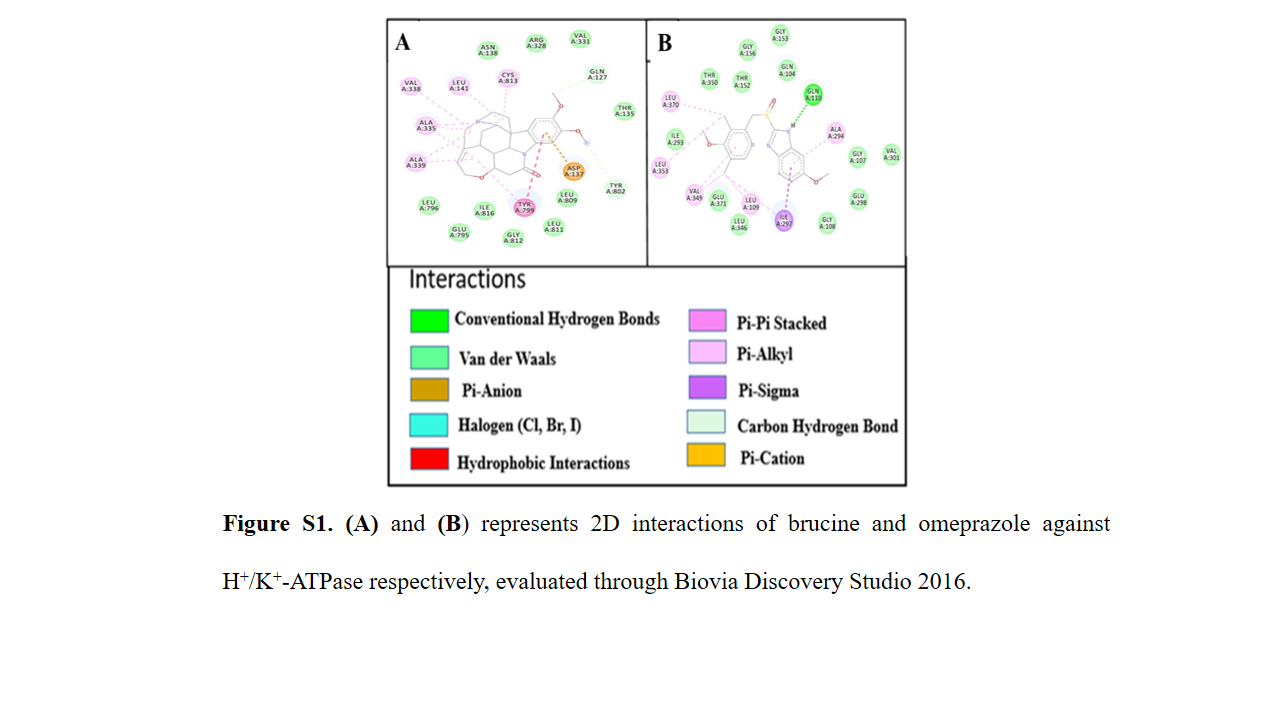

Supplement: Supplementary file 7 [file Image1.TIF]

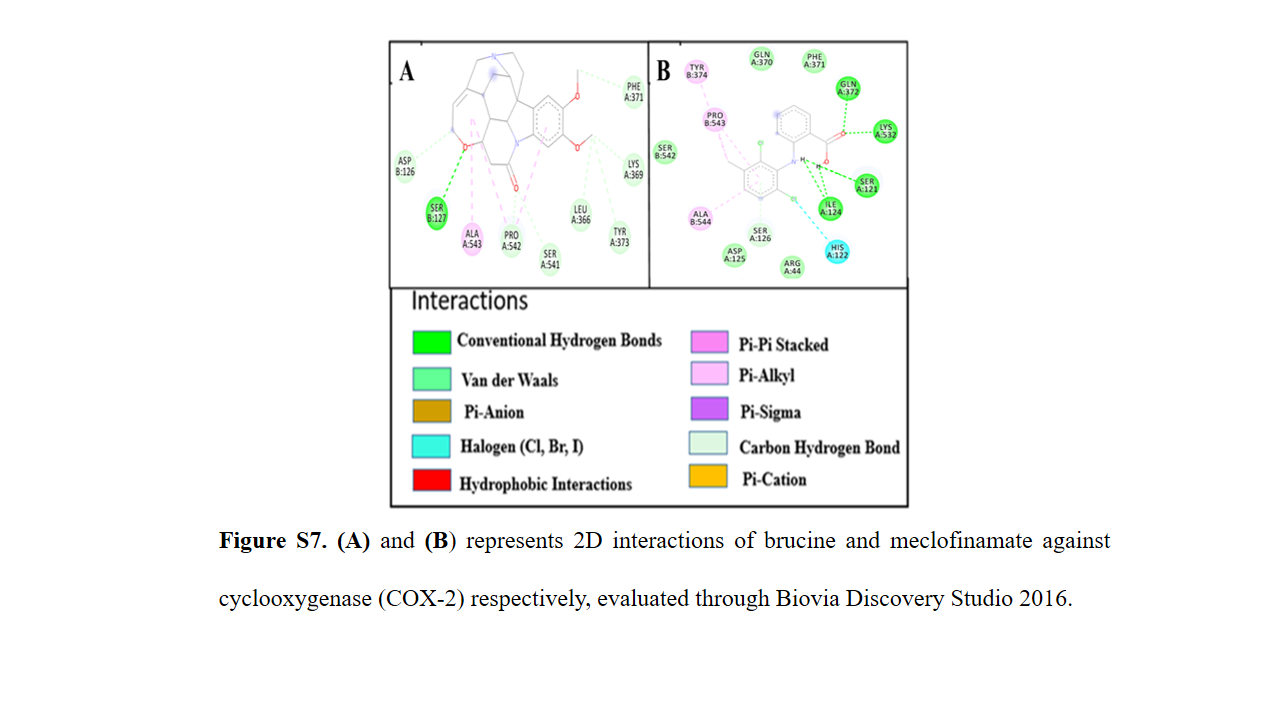

Supplement: Supplementary file 8 [file Image7.TIF]

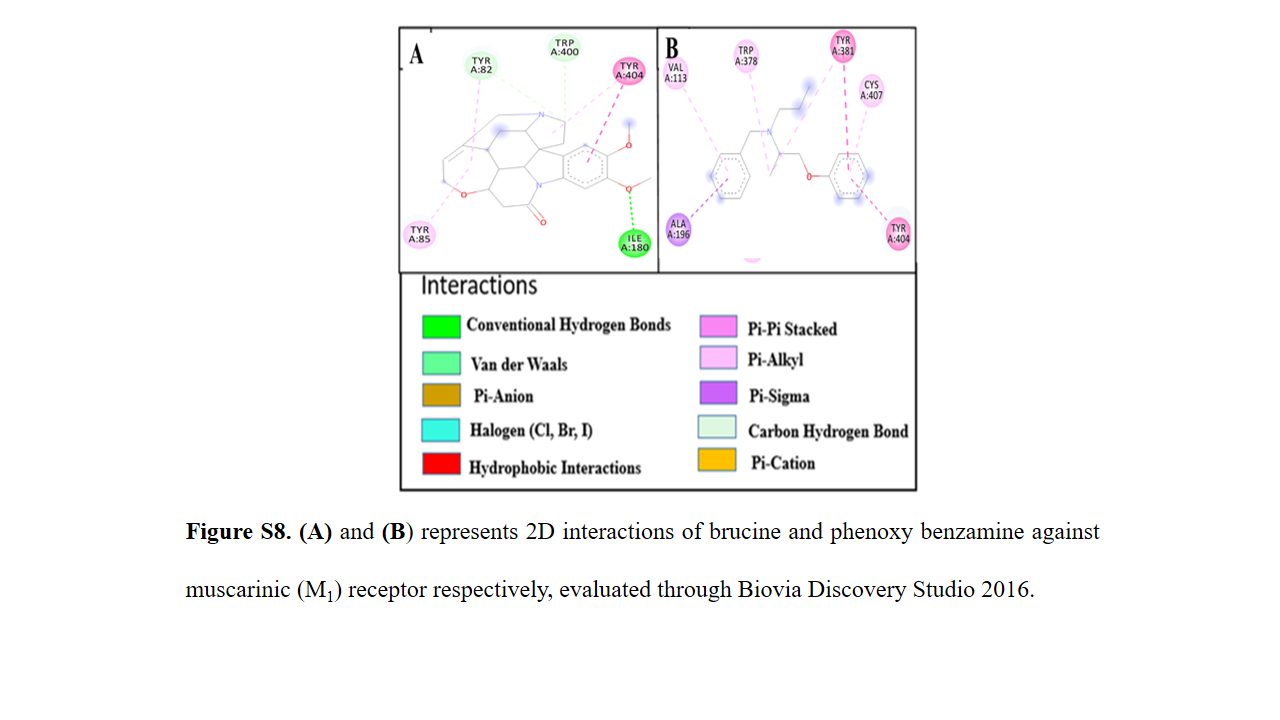

Supplement: Supplementary file 9 [file Image8.TIF]

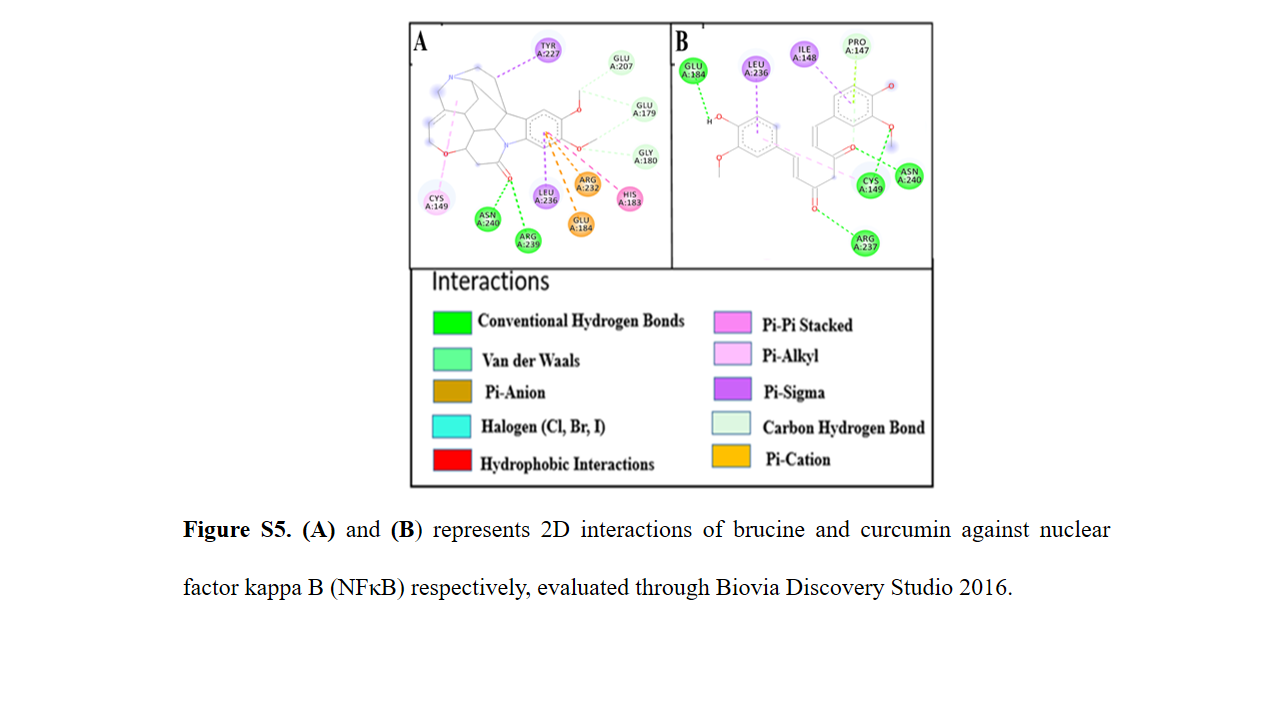

Supplement: Supplementary file 10 [file Image5.TIF]
